# Botulinum Toxin for the Management of Parkinson’s Disease: A Systematic Review

**DOI:** 10.7759/cureus.53309

**Published:** 2024-01-31

**Authors:** Ethan Slouha, Fadi Ibrahim, Sarah Esposito, Odelin Mursuli, Atbeen Rezazadah, Lucy A Clunes, Theofanis F Kollias

**Affiliations:** 1 Pharmacology, St. George's University School of Medicine, St. George's, GRD; 2 Pharmacology, St George's University School of Medicine, St George's, GRD; 3 Microbiology, Immunology, and Pharmacology, St. George's University School of Medicine, St. George's, GRD

**Keywords:** sialorrhea, dystonia, tremor, botulinum toxin, parkinson's disease

## Abstract

Parkinson’s disease (PD) is a terminal, debilitating neurodegenerative disorder typically affecting individuals over 60. It is associated with various conditions that drastically affect the patient’s quality of life (QoL). Although there is no cure for PD, its symptoms can be significantly improved and even resolved through different treatments. Mainstay treatments for PD include levodopa combined with carbidopa, dopamine agonists, and even deep brain stimulation (DBS) of the subthalamic nucleus. New treatment methods have emerged, such as botulinum toxin (BoNT), which further improve symptoms and, thus, the QoL of patients with PD. Botulinum toxin is a potent neurotoxin produced by *Clostridium botulinum* that typically causes descending paralysis by suppressing acetylcholine secretion. Serotypes used to treat various disorders include serotype A (BoNT-A) and serotype B (BoNT-B). This paper aims to evaluate the outcomes of BoNT injection on different symptoms associated with PD. An extensive review using PubMed, ScienceDirect, and ProQuest articles concerning ‘botulinum toxin and Parkinson’s disease’ was done per the Preferred Reporting Items for Systematic Reviews and Meta-Analyses (PRISMA) guidelines, resulting in 23,803 articles. After applying strict inclusion and exclusion criteria, the total number of articles was finally 41.

The results showed that movement disorders were a common occurrence in PD, consisting of tremors, dystonia, and freezing of gait (FOG), with tremors being the most common symptom. Tremors and dystonia were significantly improved following BoNT-A, correlating with significant improvements in various scales subjectively and objectively evaluating the symptoms and QoL. In contrast, FOG was not significantly improved by either BoNT-A or BoNT-B. Pain is associated with movement disorders such as PD and was the primary indication for the administration of BoNT; studies found pain and QoL were significantly improved following BoNT injection. Quality of life can also be affected by sialorrhea and overactive bladder, which often occur as the disease progresses. Injections of BoNT-A and BoNT-B were shown to significantly improve saliva production, flow rate, drooling frequency, voiding frequency, and urinary urge incontinence.

Across all studies analyzed, it is evident that BoNT may have a significant effect on improving the QoL of patients suffering from PD. While research continues to find a cure or stop the progression of PD, it remains critical to continue focusing on improving patients’ QoL. Future research should evaluate whether BoNT can be used to successfully treat other symptoms of PD, such as epiphora or constipation.

## Introduction and background

Parkinson’s disease

Parkinson’s disease (PD) is a terminal, neurodegenerative disorder affecting the basal ganglia that typically presents later in life, affecting ~1% of individuals over 60 years of age [1-3]. It was first described by James Parkinson in 1817 as “shaking palsy,” and is associated with tremors, bradykinesia, and dystonia. Other symptoms associated with PD as the disease progresses include sleep dysfunction, loss of smell, excessive salivation, mood disorder, excessive periodic limb movements during sleep, and constipation [3]. Idiopathic PD is the most common type of PD, with only 10% of individuals developing it due to genetic causes. The development of PD has been linked to herbicides, proximity to industrial plants, pesticides, altered function of alpha-synuclein, and damage to the thalamic nuclei through oxidation and the generation of free radicals [1,3]. In some rare cases, PD can occur with an early onset as young as 21 years of age, which leads to a longer duration of symptoms and a continuous hindrance to QoL [4].

The pathophysiology of PD has been associated with the loss or damage of dopaminergic neurons in the substantia nigra that project to the striatum to correlate movement, as well as the presence of Lewy bodies that form from alpha-synuclein aggregation [1-3]. A definitive diagnosis is determined postmortem, as the loss of dopaminergic neurons in the substantia nigra leads to a loss of pigmentation [3]. It has been postulated that the earliest degenerative effects of PD may occur in the myenteric plexus in the gastrointestinal tract, which progresses to the dorsal motor nucleus of the vagus nerve, then to the sleep centers in the pons, and lastly, the midbrain [2,3].

The goal of PD management is to treat the associated symptoms that arise as PD progresses, as there is no cure for the disease [1]. The mainstay treatment of PD is restoring depleted dopamine through the use of levodopa combined with carbidopa or dopamine agonists such as pramipexole and ropinirole [3]. These treatments are typically only effective for three to six years, even when paired with physical therapy to improve balance and gait. When these treatments fail, patients are usually candidates for deep brain stimulation (DBS), which requires stimulation of the subthalamic nucleus, thalamus, and globus pallidus interna. Still, DBS is fallible, as it eventually wears off and may not even work for some. Therefore, new approaches to treating symptoms associated with PD are highly desired.

Botulinum toxin

Botulinum toxin (BoNT) is a potent neurotoxin produced by *Clostridium botulinum* that causes descending paralysis when ingested [5,6]. Since the late 1970s, BoNT has been used by clinicians to treat many conditions, such as recent chronic migraine, cervical dystonia, blepharospasm, strabismus, primary axillary hyperhidrosis, adult bladder dysfunction, and spasticity, as well as by the cosmetics industry [5,6]. In treating cervical dystonia, BoNT significantly reduces neck pain and the severity of abnormal head position [6]. When treating an overactive bladder, it is injected into the detrusor to reduce urinary urgency, frequency, and incontinence. Botulinum toxin works as a potent zinc protease that binds extracellularly to receptors on cholinergic nerve terminals, cleaving one out of three soluble N-ethylmaleimide-sensitive factor attachment receptor proteins. Ultimately, this causes inhibition of acetylcholine release by intracellular presynaptic vesicles, inhibiting neurotransmitter release at the neuromuscular junction [5,6].

Essentially, BoNT provokes the weakness of striated muscles through the inhibition of alpha motor neurons found at the neuromuscular junction [5]. Peak therapeutic results usually occur around seven days following the injection. When injected, the therapeutic effectiveness of BoNT depends on its location and the type of muscle injected, with a duration ranging from three to nine months [6]. There are several FDA-approved BoNTs, including serotypes BoNT-A (e.g., onabotulinumtoxinA, abobotulinumtoxinA, and incobotulinumtoxinA) and BoNT-B (e.g., rimabotinumtoxinB (RIMA)) [5,6]. The BoNT is also associated with adverse effects that vary by location, such as bruising, pain at the injection site, edema, flu-like symptoms, blepharoptosis, eyelid ptosis, headache, and xerophthalmia. In the event of toxicity, antitoxin, vaccine, or F(ab’)2-immune fragment therapies may be used [6].

Objective

In light of the dire need to improve the QoL of patients with PD, alternative treatment methods have been theorized, applied, and evaluated. One unique treatment is the use of BoNT, which paralyzes or relaxes certain muscles that are causing significant pain, discomfort, or abnormal functions. This paper aims to evaluate the application of BoNT in treating various symptoms associated with PD, such as movement disorders (e.g., tremors and dystonia), pain associated with certain movement disorders, sialorrhea, and overactive bladder. Other symptoms associated with PD have been treated with BoNT with varying levels of success; however, insufficient research has been done to replicate and verify these results. Ultimately, this work aims to highlight BoNT and push for further evaluation of its diverse uses.

## Review

Methods

This systematic review was conducted while strictly adhering to the format presented in the Preferred Reporting Items for Systematic Reviews and Meta-Analyses (PRISMA) guidelines, per which PubMed, Science Direct, and ProQuest were used to perform an extensive inquiry of the current literature between January 1, 2003, and November 1, 2023. “Botulinum and Parkinson’s Disease” was selected as the keyword to cover all PD-associated symptoms that BoNT has been evaluated to treat.

Inclusion and Exclusion Criteria

Studies performed on humans, published between 2003 and 2023, peer-reviewed experimental and observational studies, full-text availability, and focused on the outcomes of using BoNT for symptoms associated with PD, were included in the study. Articles with no full-text availability, written in a language other than English, and duplicate publications were excluded from the study.

**Figure 1:**
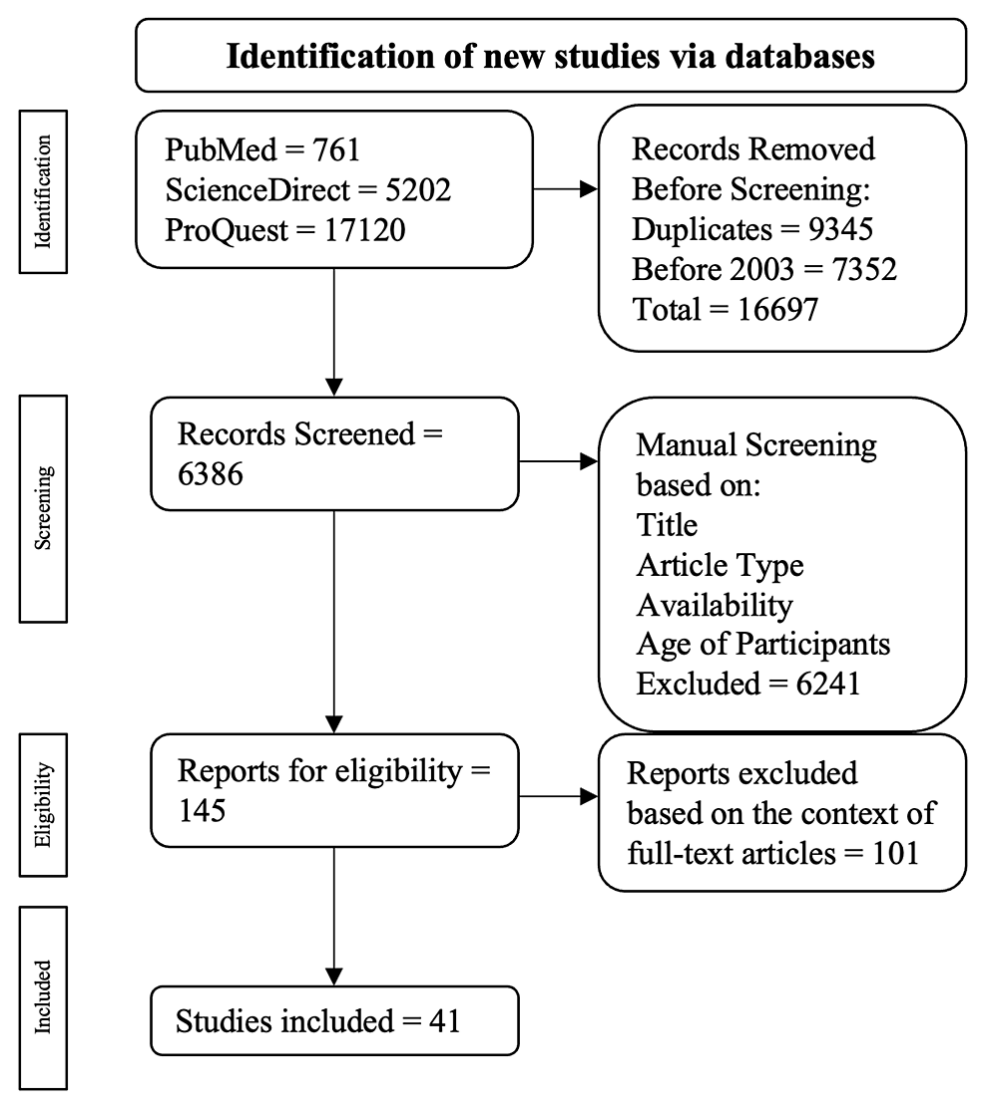
The PRISMA flow diagram depicting the algorithm employed based on the inclusion and exclusion criteria PRISMA: Preferred Reporting Items for Systematic Reviews and Meta-Analyses

The initial inquiry resulted in 23,083 publications (761 from PubMed, 5202 from ScienceDirect, and 17,120 from ProQuest). After the programmed screening which resulted in the exclusion of 9345 duplicate publications and 7352 articles published before 2003, the resulting publications were manually explored with consideration of their title, study, abstract, and full-text availability. The concluding step of the screening sequence involved the evaluation of 145 publications to determine if the text related to the topics of interest (e.g., movement disorders, sialorrhea, and overactive bladder) and further narrow down the number of publications. Ultimately, a total of 41 publications were procured for this study through the use of the criteria described (Figure 1).

Bias

Due to the significantly small sample sizes present in the majority of selected studies, there was a moderate risk of bias classification in this paper. What maintained a moderate risk, as opposed to a high risk, is that all studies explicitly stated the methods of their conducted studies, allowing for transparency. The grading of recommendation, development, and evaluation scale was used for bias assessment, which focuses on imprecision and the type of publication presented.

Results

The BoNT-A was found to significantly improve the presence and severity of tremors in patients with PD, correlating with significant improvements in the unified Parkison’s disease rating scale (UPDRS) and QoL. Dystonia is also a common occurrence in PD and can come in the form of Pisa syndrome, foot dystonia, and camptocormia, all of which were significantly improved following BoNT-A injections, as well as significant improvements in the Burke-Fahn-Marden dystonia rating scale (BFMDRS) and clinical global impression (CGI). A shuffling gait is another hallmark of PD, but most studies found that neither BoNT-A nor BoNT-B significantly improved freezing of gait (FOG), subjective CGI, or activities of daily living (ADL). Along with movement disorders comes pain, which was a main indication for starting BoNT-A; subsequent injections of BoNT-A significantly improved the numeric rating scale (NRS) for pain, subjective- and physician-related CGI, and visual analogue scale (VAS) for pain. Treatment of sialorrhea also came up in the search. It was observed that both BoNT-A and BoNT-B significantly improved sialorrhea in terms of the drooling frequency and severity scale (DFSS), VAS-drooling, production of saliva, and consistency of saliva, with no variation between them. Lastly, overactive bladder occurs in the advanced stage of PD, hindering patients’ QoL. Overactive bladders were significantly improved following injection of BoNT-A, with significant improvements in maximum cystometric capacity; stress, emptying, anatomy, protection, inhibition (SEAPI); voiding frequency; urinary urge; and incontinence.

Discussion

Treatment for Movement Disorders

Tremor is the most common symptom of PD, significantly affecting individuals’ ADL by disrupting fine motor activity. Studies have evaluated whether BoNT-A could improve tremors in patients with PD. The most frequently injected muscles were the flexor carpi ulnaris, flexor carpi radialis, extensor carpi ulnaris, extensor carpi radialis, biceps, triceps, and supinator [7]. Using kinematics to track patient movements, it was observed that there was a significant reduction in the presence and severity of tremors, which was confirmed with the severity tremor score, patient perception, and the resting tremor portion of the UPDRS [7-9]. Kinematics noted that mean finger acceleration and mean wrist root-mean-square amplitude significantly decreased following each injection [9]. However, the PD QoL score did not change substantially compared to the placebo [7,8]. In contrast, significant improvement was observed in functional activities such as pouring liquids and writing [7]. Interestingly, one study observed that the untreated limb started to show improvement at week 54, which continued throughout the study’s duration. The main adverse effects reported were weakness in the upper extremities, specifically hand and grip weakness, but this did improve throughout the studies [7-9].

Dystonia is a common complication of PD that affects different muscle groups and leads to different outcomes. Two studies observed that BoNT-A significantly lowered the BFMDRS score and improved the CGI score [10-12]. General dystonia observed in the form of Pisa syndrome involving the lateral axial was significantly improved with the administration of BoNT-A, with improvements ranging between 50% and 85.7% in lateral bending [13,14]. This effect was not observed in Ledda et al.’s study, which found no significant improvement [15]. Functional disturbances related to dystonia have also been observed to significantly improve following treatment with BoNT-A [16]. Bonanni et al. even reported that the improvements were so successful that seven patients continued BoNT-A on a three-month repeat schedule. After a year, the reduction of lateral axial dystonia was 25% to 40% better than the initial treatment period in all but one patient [14]. Lateralization of the trunk was also observed, and there was a significant decrease in lateralization, but no adverse effects were observed [17,18]. Dystonia can also lead to camptocormia (i.e., flexion of the spine), which was significantly improved with BoNT-A treatment [17,18]. Significant improvements in balance were also observed when BoNT-A was used for foot dystonia, but no significant improvements were observed in stride length or gait velocity [11]. This was because the plantar center of pressure during gait, a predictor of risk of injury, was shifted medially after treatment [11].

Gait is also significantly affected in PD, making ambulation difficult and dangerous, as falls can occur due to FOG. Studies hypothesized that BoNT-A may be used to treat abnormal gait, with one study observing significant improvement in the Berg balance scale, tandem gait, one-leg stance, timed up and go, 10-meter walk test, VAS, and UPDRS III [19]. In another study, most patients reported moderate satisfaction, with improvements in hesitation severity, duration, and subjective FOG questionnaire scores [20]. Three studies, however, observed no significant difference in FOG following BoNT-A and BoNT-B injections [21-23]. Furthermore, there was no apparent trend to indicate improvement over time in terms of subjective CGI, ADL, or the motor part of the UPDRS [22]. Although Wieler et al. observed no adverse effects following the administration of BoNT-A, Gurevich et al. [23] observed leg weakness, increased fall frequency, leg muscle pain, and dermatological changes following the BoNT-A injection [22].

Treatment for Pain

Several studies specifically evaluated pain (usually caused by dystonia) and the efficacy of BoNT in treating it. Across all studies, pain was the main indication for starting BoNT-A despite the onset of other symptoms (e.g., dystonia and sialorrhea), which included both dystonic and musculoskeletal pain [16]. Bruno et al. reported that there were no significant predictors for response to BoNT-A; however, there was a significant improvement in NRS for pain, and VAS, subjective CGI, and physician-rated CGI were significantly improved at four and 12 weeks following BoNT-A injection [11,13,14,16,19,24,25]. Individuals who suffered from dystonic pain especially saw a significant reduction in pain following BoNT-A treatment [11,24,25]. Studies reported that over 53.4% and 81% of participants saw improvements in pain and subjective CGI, respectively [16,24]. This observed improvement was maintained in one study, with 39.7% of participants reporting continuously improving outcomes [16]. Bruno et al., however, observed no improvements in physician-rated CGI, 39-item Parkinson's disease questionnaire (PDQ-39), Movement Disorder Society-sponsored revision of the unified Parkinson's disease rating scale (MDS-UPDRS), or NRS following BoNT-A treatment, whose effect did not significantly differ from that of the placebo [24]. No study reported any adverse or side effects, including cutaneous reactions, throughout the study period [14,24].

Treatment of Sialorrhea

Sialorrhea is another common complication of PD that affects the daily lives of patients, being both an inconvenience and a hazard, as choking can occur even when dysphagia is not present. Tiigimae-Saar et al. observed higher saliva levels in patients treated with levodopa than those treated with monoamine oxidase B [26]. In a study by Guidubaldi et al., both BoNT-A and BoNT-B treatments resulted in clear benefits for all patients, both subjectively and objectively [27]. An average of 78.75% of patients receiving BoNT-A experienced significant improvement in sialorrhea, with decreased saliva production [28-33]. The efficacy of BoNT-A consistently improved throughout one study, reaching 100% at the end of the study period [32]. Significant decreases in VSA-drooling and DFSS (22.21% and 36.5%, respectively) were also observed [29,31,34,35]. Within the DFSS, severity and frequency decreased significantly by 37.43% and 12.28%, respectively [31]. Self-reported drooling intensity also decreased significantly, with one study reporting a 20.83% decrease [26,35]. The UDPRS and UDPRS-ADL scores were also significantly improved following BoNT-A treatment, with Lagalla et al. reporting that 88% of patients were willing to receive repeated injections [29,34].

One study compared the effects of BoNT-A given at two different injection sites: the submandibular and the parotid glands. The DFSS and social consequences scale scores were significantly reduced in patients with submandibular injections [36]. However, there was no significant difference in UPDRS-drooling [36]. When injecting BoNT-A into the parotid glands, there was no significant difference in DFSS, UPDRS-drooling, or social consequences scale scores [36]. However, DFSS was significantly reduced in patients with submandibular injections of BoNT-A compared to those injected in the parotid gland.

As mentioned, BoNT-A and BoNT-B have both been used to successfully treat sialorrhea. In studies on BoNT-B treatment specifically, there was a significant improvement in DFSS scores over time, as well as in global impression of change, CGI-drooling, VAS-drooling, and drooling rating scale (DRS) scores [37-41]. One study reported that DFSS decreased by 31.1% and the DRS decreased by 35.1% following BoNT-B treatment [41]. Swallowing was also improved compared to the placebo group [37]. Lagalla et al. reported that the effect duration of BoNT-B was 10.2 weeks on average, which was significantly longer than the placebo group [40]. The efficacy of BoNT-B in treating sialorrhea has also been evaluated, and it was observed that the unstimulated saliva frequency rate (USFR) significantly decreased following treatment, with an improvement in CGI [39]. When comparing BoNT-A and BoNT-B, BoNT-B treatments reduced cotton roll weights more effectively at one-week follow-up, but this trend did not continue throughout the study [27]. Furthermore, compared with BoNT-A, BoNT-B treatment had a significantly shorter onset of action.

Although adverse effects were rarely reported concerning movement and pain, several side effects were noted in sialorrhea treated with BoNT-A and BoNT-B. The adverse effects directly related to the injection procedure were minimal and did not show any significant differences between BoNT-A and BoNT-B treatments [27]. A common side effect of both BoNT treatments was dry mouth resulting from the thickening of saliva [26,27,36,37,39,41]. However, dry mouth was reported at a rate that was three times as high after submandibular injections than after parotid injections [36]. Concerning BoNT-A specifically, some patients experienced local edema, mild and transient difficulties in swallowing, and decreased chewing acts [28,29,31,33,36]. Two studies, however, reported that no adverse effects were experienced throughout the study period [30,32]. Regarding BoNT-B, neck pain, dysphagia, dental carries, and mild weakness of chewing were observed but were not common [39-41].

Treatment of Overactive Bladder

Overactive bladder occurs late in PD, with urodynamic studies revealing that most PD patients have detrusor muscle overactivity, specifically with low uninhibited detrusor contraction (UDC) at first volume and high UDC at maximum pressures. Following treatment with BoNT-A, conflicting results were observed regarding UDC at first volume; Giannantoni et al. first observed a significant decrease in UDC at first volume, but two years later, with a different sample, they instead observed a significant increase [42,43]. However, in both studies, there was a significant increase in maximum cystometric capacity, representing bladder function [42,43]. Potential predictors of BoNT-A treatment success include a higher UPDRS, lower Schwab and England scores, lower preoperative maximum cystometric capacity, a longer duration of PD, and a lower preoperative first bladder filling sensation compared to those not treated [44].

Concerning symptoms, significant improvements were observed in SEAPI scores and functional bladder capacities following BoNT-A injection [44,45]. There was also a significant decrease in voiding frequency during both daytime and nighttime, as determined subjectively with a voiding diary [42,43,45,46]. Urinary urge and incontinence severity also decreased significantly, which was observed to be due to a decrease in the mean pressure of uncontrolled bladder contractions [42-47]. Giannantoni et al. found that post-void residual volume increased after one month of treatment but significantly decreased during the remainder of the study [42]. During and after the treatment period, patients reported a significant improvement in their QoL [42,43,45]. No adverse effects (e.g., urinary tract infections, urinary retention, or systemic adverse effects) were reported [42,43,46]. Table 1 summarizes the studies reviewed in this article.

**Table 1:** Summary of articles assessed in this review DFSS: Drooling frequency severity scale; UPDRS: Unified Parkison’s disease rating scale; BoNT-A: Botulinum toxin A; PD: Parkinson's disease; VAS: Visual analogue scale; VAS-FD: Visuo-analogic ratings of familial distress; VAS-SD: Visuo-analogic ratings of social distress; LAD: Lateral axial deviation; DRS: Drooling rating scale; BoNT-B: Botulinum toxin B (rimabotulinumtoxinB); CGI:  Clinical global impression; CGI-C: Clinician global impression of change; QoL: Quality of life; FOG: Freezing of gait; UUI: Urinary urge incontinence; FOGQ: Freezing of gait questionnaire; TUG: Timed up and go; BFMDRS: Burke-Fahn-Marden dystonia rating scale; USFR: Unstimulated salivary flow rate; USS: Urgency severity score; BBT: Blindfolded balance training; TDDS: Transdermal drug delivery system; GAS: Goal attainment scale; DFSS: Drooling frequency and severity scale; RIMA: RimabotulinumtoxinB

Article	Authors	Country	Design & Study Population	Findings	Conclusion
1	Samotus et al., 2017 [8]	Canada	Phase II pilot study (n=52)	After BoNT-A injections, functional ability and ratings of tremor severity significantly improved throughout the study period. There was a significant 70% reduction in tremor amplitude.	BoNT-A injections significantly lowered tremor severity in PD patients while simultaneously increasing their functional ability.
2	Mittal et al., 2017 [9]	USA	RCT (n=30)	There was a significant improvement in tremor severity and resting tremor following BoNT-A treatment, as well as action/postural tremor. This correlated with patients’ perception of improvement, which was significantly increased.	BoNT-A provided significant relief for PD patients who experience tremors.
3	Rahimi et al., 2015 [10]	USA	Open-label study (n=28)	Both maximal grip strength and mean finger acceleration decreased in injected muscles by participants. Kinematics also showed reduced tremor severity at each arm joint.	Although adverse effects (e.g., decreased grip strength) may be present in some patients, BoNT-A may improve tremors in PD patients.
4	Gupta et al., 2016 [11]	Australia	Pilot study (n=6)	Three weeks following BoNT-A injection, there was a significant improvement in pain, dystonia, UPDRS, gait velocity, and cadence. Most patients had significant improvement in the TUG test and GAS goals.	BoNT-A can significantly improve foot dystonia and limb functions in PD patients.
5	Huang et al., 2021 [12]	Canada	Open-Label Study (n=6)	Injection with BoNT-A led to significant improvement in BFMDRS, VAS, TUG, BBT, and 3D gait analysis.	BoNT-A injections were found to effectively alleviate foot muscle spasms, reduce pain associated with dystonia in PD, and improve balance.
6	Patterson et al., 2016 [13]	USA	Cross-sectional study (n=144)	Following BoNT-A injections, CGI scores improved significantly, but a small percentage of patients experienced dystonia.	BoNT-A may be a safe and effective treatment of cervical dystonia associated with PD.
7	Artusi et al., 2019 [14]	Italy	Pilot study (n=13)	84.6% of patients responded to BoNT-A injections with an average reduction of trunk bending in 40%. Discomfort/pain was also significantly improved, with a 52.2% reduction in VAS.	Pisa syndrome, when associated with PD, can be well treated with BoNT-A injections in the paraspinal and non-paraspinal axial muscles.
8	Bonanni et al., 2007 [15]	Italy	Prospective crossover trial (n=1400)	There was an improvement in goniometric grading, TDDS, and VAS scores in the majority of patients, suggesting that BoNT-A may be a successful treatment option.	BoNT-A treatment appeared to be well-tolerated and effective for alleviating LAD in patients with L-dopa-responsive PD.
9	Ledda et al., 2023 [16]	Italy	RCT (n=14)	BoNT did not lead to a significant improvement in lateral trunk flexion.	BoNT had varying effects in axial regions.
10	Bruno et al., 2016 [17]	Canada	Retrospective cohort study (n=160)	The main indication of BoNT treatment was pain, which was significantly improved following injection.	BoNT may prove to be highly beneficial in treating pain associated with PD.
11	Tassorelli et al., 2014 [18]	Italy	RCT (n=26)	There was a significant improvement in range of motion and static postural alignment following BoNT-A and rehabilitation. BoNT-A led to a significant reduction in pain scores compared to the placebo.	Patients with PD experiencing Pisa syndrome may experience beneficial effects following BoNT-A injections.
12	Todo et al., 2018 [19]	Japan	Prospective open-label study (n=6)	Patients injected with BoNT-A in their bilateral external obliques experienced significant improvement in their camptocormia within two weeks of injection.	BoNT-A treatment for patients with camptocormia resulting from severe PD seemed to be effective.
13	Lindholm et al., 2016 [20]	Sweden	Pilot study (n=10)	BoNT-A injections led to significant improvements in dynamic, functional, and standing balance, as well as a decrease in the intensity of distress.	BoNT-A significantly improves gait and balance for those with PD and striatal foot deformities.
14	Neshige et al., 2022 [21]	Japan	Pilot study (n=5)	The majority of patients experienced moderate satisfaction with improvement in their freezing of gait one week following BoNT-A injections. This effect lasted more the one month.	BoNT-A injection into the psoas muscle may be a good treatment option for FOG.
15	Fernandez et al., 2004 [22]	USA	RCT (n=14)	Injection of 5000 U of BoNT-B into one gastrocnemius-soleus complex did not significantly improve FOG in patients with idiopathic PD.	Subsequent investigations are imperative to ascertain the potential efficacy of BoNT-A in addressing FOG in PD.
16	Gurevich et al., 2007 [23]	Israel	Double-blind placebo-controlled pilot study (n=11)	Neither group (BoNT-A injection and saline injection) demonstrated a positive effect on FOG severity when injected into the calf muscles.	Injection of BoNT-A into the calf muscle in PD patients did not result in an improvement in FOG and could potentially lead to an increased risk of falls, possibly due to leg weakness.
17	Wieler et al., 2005 [24]	Canada	Randomized double-blind crossover study (n=12)	There was no difference in FOGQ, UDPRS, or TUG test scores following the BoNT-A injection.	BoNT-A may not be effective at treating FOG in PD patients.
18	Bruno et al., 2018 [25]	Canada	Randomized double-blind crossover study (n=12)	BoNT-A injections for painful limbs in advanced PD revealed a mild reduction in pain after four weeks compared to the placebo, and no significant adverse events in patients with advanced PD.	Despite the safety of BoNT-A in patients with limb pain and advanced PD, the study did not demonstrate a significant therapeutic effect compared to the placebo.
19	Rieu et al., 2018 [26]	France	RCT (n=45)	There was a significant improvement in CGI following the BoNT-A injection. Although pain and dystonia were significantly reduced, there was no difference between the BoNT-A and placebo groups.	BoNT-A injections were effective at improving pain and dystonia associated with planar flexion and toe dystonia in PD patients.
20	Tiigimae-Saar et al., 2018 [27]	Estonia	Prospective clinical trial (n=38)	BoNT-A injections significantly improved the salivary flow rate with no change in the composition of saliva.	BoNT-A is an effective modality to treat sialorrhea for PD without changing the composition of saliva.
21	Guidubaldi et al., 2011 [28]	Italy	Prospective, randomized, double-blind, crossover pilot study (n=14)	Both BoNT-A and BoNT-B treatments resulted in improvements in both subjective and objective measures of drooling. However, BoNT-B had a significantly shorter onset of action compared to BoNT-A (3.4 days less). The duration of benefit was similar for both treatments. The only notable side effect was a change in saliva thickness.	Both BoNT-A and BoNT-B were effective and safe in controlling sialorrhea. BoNT-B had a quicker onset of action, and the duration of benefit was comparable to that of BoNT-A. A cost analysis, based on the doses used in the study, favored BoNT-B treatment.
22	Gomez-Caravaca et al., 2015 [29]	Spain	Retrospective cohort study (n=53)	65.22% of patients had significant improvement of their sialorrhea after BoNT-A treatment. The effect had an average duration of 10.06 days.	BoNT-A is a safe and effective treatment for sialorrhea in PD.
23	Lagalla et al., 2006 [30]	Italy	Double-blind RCT (n=32)	Subjects treated with BoNT-A showed a reduction in drooling frequency, saliva production, and familial and social disability.	BoNT-A injection into the parotid gland can be considered the treatment of choice for sialorrhea in PD patients.
24	Mancini et al., 2003 [31]	Italy	RCT (n=20)	The secretion of saliva was significantly improved following BoNT-A injection of the parotid and submandibular glands.	BoNT-A injection was effective and had no side effects in treating sialorrhea in PD patients.
25	Nobrega et al., 2007 [32]	Brazil	Retrospective cohort study (n=21)	DFSS was reduced significantly in patients using BoNT-A	Patients with PD taking levodopa may experience decreased sialorrhea when using BoNT-A.
26	Sen et al., 2015 [33]	Turkey	Retrospective cohort study (n=16)	BoNT-A injections showed a significant decrease in DFSS with 100% efficacy.	IBoNT-A improved sialorrhea without any significant side effects in PD patients.
27	Svetel et al., 2009 [34]	Serbia	Quasi-experimental study (n=19)	68% of patients who received BoNT-A injection in the parotid gland reported beneficial effects, and sialorrhea scores decreased significantly.	BoNT-A may be an effective treatment for sialorrhea.
28	Alagoz et al., 2020 [35]	Turkey	Retrospective cohort study (n=21)	There was a significant decrease in DFSS and UPDRS at all time intervals in patients who received BoNT-A.	BoNT-A significantly decreased sialorrhea in patients with PD and was safe, simple, and tolerable.
29	Moller et al., 2011 [36]	Denmark	Retrospective cohort study (n=15)	There was a significant decrease in saliva production in patients using BoNT-A.	BoNT-A was useful in treating sialorrhea in patients with PD.
30	Kalf et al., 2007 [37]	Netherlands	Pilot study (n=17)	BoNT-A injections in the submandibular group of patients with PD showed significant improvement compared to the parotid group. The DFSS and social consequences scale were significantly improved in the submandibular group but not in the parotid group.	Injection of BoNT-A into the submandibular glands appeared to be a promising approach for managing sialorrhea in PD patients.
31	Chinnapongse et al., 2011 [38]	USA	RCT (n=54)	BoNT-B significantly improved DFSS and USFR, which was sustained throughout the study period. However, the most common adverse effect was dry mouth, which was seen in 31% of patients.	Intraglandular injection of BoNT-B was well-tolerated and significantly improved sialorrhea in patients with PD, suggesting its robust efficacy.
32	Contarino et al., 2007 [39]	Italy	RCT (n=21)	One week following BoNT-B injections, cotton roll weights and subjective evaluations significantly improved, correlating with a reduction in sialorrhea.	BoNT-B significantly improved sialorrhea in PD patients.
33	Isaacson et al., 2020 [40]	USA	RCT (n=176)	Treatment with RIMA significantly decreased USFR. The CGI-C scores also improved significantly.	RIMA may be an effective treatment for reducing USFR and improving CGI-C.
34	Lagalla et al., 2009 [41]	Italy	Double-blind RCT (n=36)	One month following BoNT-B treatment, there was a significant improvement in DFSS, VAS-FD, and VAS-SD, with an objective reduction in saliva. There was also a significant improvement in GIS.	BoNT-B is a safe and effective tool for managing PD-related sialorrhea, providing long-lasting relief from this debilitating symptom.
35	Ondo et al., 2004 [42]	USA	Retrospective cohort study (n=16)	There was a significant decrease in DRS and DFSS in patients treated with BoNT-B.	Sialorrhea in patients with PD can be reduced with BoNT-B use.
36	Giannantoni et al., 2011 [43]	Italy	Open-label study (n=8)	BoNT-A injections in the intradetrusor muscle led to a decrease in both daytime and nighttime urinary frequency, a reduction in urinary incontinence episodes, and improved QoL for all patients. Urodynamic assessments indicated an increased maximum cytometric capacity, meaning improved bladder function.	BoNT-A significantly improved overactive bladder symptoms in PD patients who were not responsive to anticholinergic medications. These improvements lasted for at least six months, suggesting that this treatment may be viable for managing detrusor overactivity.
37	Giannantoni et al., 2009 [44]	Italy	Prospective clinical trial (n=6)	Following injection with BoNT-A day and night-time urinary frequency significantly decreased. There was also a significant increase in QoL. No patients reported any urinary urgency or UUI following injections.	BoNT-A injection into the detrusor muscle proved to be an effective and safe treatment for refractory overactive bladder symptoms and detrusor overactivity linked to PD.
38	Vurture et al., 2018 [45]	USA	Retrospective cohort study (n=24)	79.2% of patients reported improvement of their overactive bladder, and 29.1% saw a complete resolution of UUI following BoNT-A injection.	BoNT-A proved to be highly effective at treating overactive bladder.
39	Kulaksizoglu et al., 2010 [46]	Turkey	Open-label study (n=16)	Following BoNT-A injection, voiding frequency and urinary incontinence significantly improved. QoL from the perspective of primary caregivers and patients also significantly improved.	Intravesicular BoNT-A injections were an effective treatment option with local action and no observed central nervous system side effects in PD patients.
40	Anderson et al., 2014 [47]	USA	Open-label study (n=20)	There was a moderate to marked improvement of neurogenic bladder symptoms following BoNT-A, with a 50% decrease in incontinence.	BoNT-A can be a safe and effective treatment for neurogenic bladder in patients with PD, and can be done with an office cystoscopy.
41	Jiang et al., 2014 [48]	Taiwan	RCT (n=200)	Patients receiving BoNT-A had significant improvement in USS, and UUI, and post-void residual volume increased significantly three months after therapy.	BoNT-A may prove to be an effective treatment for overactive bladders in PD patients.

One strength of this review is the variety of countries from which studies were found, increasing its representativeness of the general population. One limitation of this study is its minimal research involving bladder control and movement difficulties in PD. Several studies evaluated movement but focused instead on different aspects, with tremor being the most evaluated condition for BoNT use. Another limitation concerns the sample size of some of these studies, as many were initial randomized controlled trials concerning BoNT. It is also important to note that there have been studies on other applications of BoNT; however, they suffer from insufficient replication, forcing us to exclude them from this study.

## Conclusions

Parkinson's disease is a debilitating and terminal neurodegenerative disorder that typically affects older individuals but can also exhibit early onset, where QoL is significantly hindered. Multiple symptoms may present throughout the duration of the disease, such as tremors, bradykinesia, pain, sialorrhea, an overactive bladder, and dementia, with tremors being the most common symptom. Movement disorders in PD are frequently treated with DBS, and this mode is highly effective. Botulinum toxin is a neurotoxin that typically causes descending paralysis and has most commonly been used for cosmetics, overactive bladders, and even hyperhidrosis. However, recently, physicians have started to evaluate the effects of BoNT when treating different PD symptoms.

While researchers continue to develop a cure for PD, finding treatment options for its associated symptoms and improving patients’ QoL is important. Botulinum toxin has been found to have many applications in treating symptoms associated with PD with great success. A few studies evaluated the effects of BoNT-A, BoNT-B, and RIMA, but only one compared these options. As more studies evaluate the success of BoNT for symptoms of PD, there should be further investigation of the three above-mentioned BoNTs, including those not included but proven to be successful. Other studies have evaluated the use of BoNT-A for epiphora as well as constipation associated with PD. However, while demonstrating some improvement, these studies are drastically limited due to a lack of repetition and reproduction, and therefore they were not included in this study. Further research should explore these specific applications and be able to reproduce the current success reported, thus allowing for further improvement of symptoms and conditions associated with PD.
